# The Autophagosomes Containing Dengue Virus Proteins and Full-Length Genomic RNA Are Infectious

**DOI:** 10.3390/v13102034

**Published:** 2021-10-09

**Authors:** Shan-Ying Wu, Yu-Lun Chen, Ying-Ray Lee, Chiou-Feng Lin, Sheng-Hui Lan, Kai-Ying Lan, Man-Ling Chu, Pei-Wen Lin, Zong-Lin Yang, Yen-Hsu Chen, Wen-Hung Wang, Hsiao-Sheng Liu

**Affiliations:** 1 Department of Microbiology and Immunology, School of Medicine, College of Medicine, Taipei Medical University, Taipei 110, Taiwan; shanyingwu@tmu.edu.tw (S.-Y.W.); cflin2014@tmu.edu.tw (C.-F.L.); 2 Graduate Institute of Medical Sciences, College of Medicine, Taipei Medical University, Taipei 110, Taiwan; 3 Department of Microbiology and Immunology, College of Medicine, National Cheng Kung University, Tainan 701, Taiwan; alice8255@yahoo.com.tw; 4 Department of Microbiology and Immunology, College of Medicine, Kaohsiung Medical University, Kaohsiung 807, Taiwan; yingray.lee@gmail.com; 5 Core Laboratory of Immune Monitoring, Office of Research & Development, Taipei Medical University, Taipei 110, Taiwan; 6 Center of Infectious Diseases and Signaling Research, National Cheng Kung University, Tainan 701, Taiwan; 7 Department of Life Sciences and Institute of Genome Sciences, National Yang Ming Chiao Tung University, Taipei 112, Taiwan; shlan@ym.edu.tw (S.-H.L.); ellalan@ym.edu.tw (K.-Y.L.); in049729@gmail.com (Z.-L.Y.); 8 Center for Cancer Research, Graduate Institute of Clinical Medicine, College of Medicine, Kaohsiung Medical University, Kaohsiung 807, Taiwan; c_manling@yahoo.com.tw (M.-L.C.); peiwen349@gmail.com (P.-W.L.); 9 Division of Infectious Disease, Department of Internal Medicine, Kaohsiung Medical University Hospital, Kaohsiung Medical University, Kaohsiung 807, Taiwan; d810070@kmu.edu.tw (Y.-H.C.); bole0918@gmail.com (W.-H.W.); 10 Sepsis Research Center, Center of Tropical Medicine and Infectious Diseases, Graduate Institute of Medicine, School of Medicine, Kaohsiung Medical University, Kaohsiung 807, Taiwan; 11 Department of Biological Science and Technology, College of Biological Science and Technology, National Yang Ming Chiao Tung University, HsinChu 300, Taiwan; 12 Institute of Medical Science and Technology, National Sun Yat-sen University, Kaohsiung 804, Taiwan; 13 Master of Science Program in Tropical Medicine, College of Medicine, Kaohsiung Medical University, Kaohsiung 807, Taiwan

**Keywords:** dengue virus, HMGB1, secretory autophagy, infectious autophagosome

## Abstract

Autophagic machinery is involved in selective and non-selective recruitment as well as degradation or exocytosis of cargoes, including pathogens. Dengue virus (DENV) infection induces autophagy that enhances virus replication and vesicle release to evade immune system surveillance. This study reveals that DENV2 induces autophagy in lung and liver cancer cells and showed that DENV2 capsid, envelope, NS1, NS3, NS4B and host cell proinflammatory high mobility group box 1 (HMGB1) proteins associated with autophagosomes which were purified by gradient centrifugation. Capsid, NS1 and NS3 proteins showing high colocalization with LC3 protein in the cytoplasm of the infected cells were detected in the purified double-membrane autophagosome by immunogold labeling under transmission electron microscopy. In DENV infected cells, the levels of capsid, envelope, NS1 and HMGB1 proteins are not significantly changed compared to the dramatic accumulation of LC3-II and p62/SQSTM1 proteins when autophagic degradation was blocked by chloroquine, indicating that these proteins are not regulated by autophagic degradation machinery. We further demonstrated that purified autophagosomes were infectious when co-cultured with uninfected cells. Notably, these infectious autophagosomes contain DENV2 proteins, negative-strand and full-length genomic RNAs, but no viral particles. It is possible that the infectivity of the autophagosome originates from the full-length DENV RNA. Moreover, we reveal that DENV2 promotes HMGB1 exocytosis partially through secretory autophagy. In conclusion, we are the first to report that DENV2-induced double-membrane autophagosomes containing viral proteins and full-length RNAs are infectious and not undergoing autophagic degradation. Our novel finding warrants further validation of whether these intracellular vesicles undergo exocytosis to become infectious autophagic vesicles.

## 1. Introduction

Dengue is an arboviral disease caused by the dengue virus (DENV), which is transmitted to humans by mosquitoes of the *Aedes* family. DENV is a re-emerging virus worldwide, possibly due at least in part to global warming. Therefore, there is an urgent need to develop effective vaccines and therapeutic strategies to combat this growing threat. DENV consists of four serotypes and is an enveloped positive-strand RNA virus belonging to the Flaviviridae family. Each of the four serotypes can cause disease symptoms ranging from mild dengue fever to severe dengue, including hemorrhagic fever and shock syndrome. The genome of DENV is over 11 kb and contains 3 structural and 7 nonstructural genes [1,2]. The capsid protein is essential in virus assembly as it packages de novo synthesized RNA to form a nucleocapsid [3]. Precursor membrane glycoprotein (prM) is important in the formation and maturation of the viral particle. During virus maturation, prM is cleaved into pr and M fragments, which occurs in the acidic post-Golgi vesicles, by cellular protease furin [4]. The envelope protein located on the surface of the mature viral particle participates in the initial attachment of this particle to the host cell and regulation of the inflammatory response [5,6]. Non-structural protein 1 (NS1) exists as multiple oligomeric forms and is found in various cellular locations. Intracellular NS1 plays an essential role in virus replication and colocalizes with double-strand RNA (dsRNA) together with other components of replication complexes [7]. The NS3 protein plays a role as it encodes RNA helicase and NTPase/RTPase [8,9]. NS4B is involved in blocking IFN signaling and inducing autophagosome formation [10]. All of these structural and non-structural proteins of DENV play diverse roles in DENV pathogenesis. 

Autophagy is functionally classified as conventional degradative autophagy and unconventional secretory autophagy. The former regulates cellular metabolism to protect the cell from stresses caused by damage through degradative machinery [11]. The latter is a poorly understood process. The double-membrane autophagosome-like vesicles along with the recruited cargoes fuse with the plasma membrane instead of lysosomes for exocytosis. Traditionally, the cargoes in the cell are delivered through the conventional endoplasmic reticulum-Golgi apparatus-plasma membrane pathway. In contrast, the proteins without secretory signaling sequences are transported to the extracellular environment by a different mechanism. Secretory autophagy affects the secretion of diverse cargoes ranging from cytokines (IL-1, IL-6, IL-18, CXCL8 and HMGB1), granule contents, ATP and mitochondria to viral particles, including poliovirus, coxsackievirus B, Enterovirus 71 (EV71) and bacteria [12,13,14]. Autophagy-related vesicles (LC3+) are able to transport the mature infectious dengue particles, thereby allowing virus transmission while avoiding antibody neutralization [15]. A recent report demonstrated that a Lyn-dependent exit route of DENV in LC3+ secretory organelles enable them to evade circulating antibodies and might affect tissue tropism [16]. Aberration autophagy-dependent secretion may progress to the development of various diseases, including pathogen infection and neurodegeneration, as well as innate and adaptive immune responses. 

We previously reported that DENV2 and EV71 infection-induced autophagy promotes viral replication and pathogenesis [17,18,19,20,21,22]. DENV2 infection activates the autophagy machinery to form autophagosomes. The marker of autophagosome LC3 protein colocalized with dsRNA, DENV-NS1 and ribosomal protein L28, indicating that the DENV replication machinery is located on the autophagic vesicles [23]. Furthermore, DENV infection induced-autophagy also regulates cellular mechanisms, such as lipid metabolism and DENV2 induced-autophagy selectively engulfs the lipid droplets followed by the release of free fatty acids, which are converted to β-oxidation to produce ATP and promote DENV replication [24]. Furthermore, we previously reported that DENV2-induced glycolysis and ER stress increases autophagy activity, viral replication and pathogenesis through different signaling pathways both in vitro and in vivo [17,18]. Moreover, DENV NS4A protein can also induce autophagy through the activation of unfolded protein response (UPR) [25]. In addition, DENV can activate the AMP kinase-mTOR axis to stimulate proviral lipophagy [26]. These findings imply that autophagy degradative machinery plays a pivotal role in DENV replication. However, emerging evidence reveals that autophagy also participates in extracellular vesicle trafficking of specific proteins and molecules [14,27,28]. For example, inflammatory cytokine IL-1β and HMGB1 are released by secretory autophagy mediated by Rab8a (a small GTPase) in mammalian cells [29]. Nevertheless, current evidence on the mechanisms of flavivirus secretion is limited.

This study reveals that DENV2 structural and non-structural proteins, as well as viral genomic RNAs, are in the purified autophagosomes which are infectious. Based on the findings of Li et al., mature DENV exits the cell through the Lyn-dependent (a src family tyrosine kinase) secretory autophagy machinery, implying that the intracellular infectious autophagosomes carrying DENV proteins and genomic RNAs may use the similar pathway to exit the cargoes. 

## 2. Materials and Methods

### 2.1. Cell Culture

A549 (Human lung carcinoma), Vero (Monkey kidney), BHK-21 (Baby hamster kidney) and Huh7 (Human hepatoma) cells were cultured in Dulbecco’s Modified Eagle’s Medium (DMEM) (Invitrogen) at 37 °C with 5% CO_2_. All of these cells were supplemented with 10% fetal bovine serum (FBS; Biological Industries, Kibbutz Beit Haemek, Israel) and penicillin/streptomycin with 10,000 units of penicillin and 10 mg of streptomycin/mL (Sigma, Saint Louis, MO, USA). 

### 2.2. Dengue Virus

Vero cells were maintained at 37 °C in DMEM supplemented with 10% FBS and penicillin/streptomycin. DENV2 (16,681 strain) was maintained in Vero cells with DMEM containing 2% FBS and cultured at 37 °C for 4 to 5 days. The cultured media were collected and frozen at −70 °C. The virus titer was determined in BHK-21 cells by plaque assay [30]. For the virus infection experiment, cells were infected with DENV2 at 37 °C for 2 h. The cells were then incubated at 37 °C in the normal medium with or without specific treatment.

### 2.3. Immunoblotting

The cell lysates were collected from the cells after various treatments and total protein was extracted with the lysis buffer [1 mL RIPA, 4 μL Na_3_SVO_4_ (0.5 M), 20 μL EGTA (0.1 M), 10 μL PMSF (0.1 M), 5 μL aprotinin (2 mg/mL) and 5 μL leupeptin (2 mg/mL) and 2 μL EDTA (0.5 M)]. Total protein was separated by SDS-polyacrylamide gel and transferred to polyvinylidene difluoride membranes (EMD Millipore, Burlington, MA, USA) in the transfer buffer (0.025 M Tris-Base, 0.2 M Glycin) at 70 volts for 3 h using electroblotting (Amersham Pharmacia Biosciences Corp., Piscataway, NJ, USA). Membranes were blocked with 5% skim milk for 1 h. The primary antibodies LC3B (MBL), capsid (GeneTex, Irvine, CA, USA), envelope (GeneTex), NS1 (Sigma), NS3 (GeneTex) and NS4B (GeneTex) were used to detect specific proteins. The membrane was rinsed with enhanced chemiluminescence (ECL) (WBKLS0500; Millipore) and exposed by BioSpectrum AC (101-206-009; UVP, Upland, CA, USA).

### 2.4. Immunofluorescence Staining

The A549 cells were seeded into 6-well plates containing cover glasses (22 mm × 22 mm, Deckglaser, Paul Marienfeld GmbH & Co. KG, Lauda-Königshofen, Germany) and incubated at 37 °C for 24 h. The cells were infected by DENV2 for 2 h and then were replaced with 10% FBS DMEM [31]. The cells were fixed in 3.7% formaldehyde for 30 min. After washing with phosphate-buffered saline (PBS), the cells were incubated for 30 min in 0.1% Triton X-100 in PBS. The primary antibodies were added to the plate and incubated at 4 °C overnight. The primary antibodies LC3B (MBL), capsid (GeneTex), envelope (GeneTex), NS1 (Sigma), NS3 (GeneTex) and NS4B (GeneTex) were used to detect specific proteins. Hoechst (5 mg/mL; Sigma) was used at a dilution of 1:500 in PBS to stain the nucleus. The secondary antibodies: Anti-Mouse IgG Antibody (FITC or PE conjugate) and anti-Rabbit IgG Antibody (FITC or PE conjugate) were used and the mounting media is glycerol gelatin aqueous slide mounting medium (Sigma-Aldrich). The fluorescent change of the cells was detected under a multi-photon confocal microscope (Olympus, FV1000MPE, Tokyo, Japan).

### 2.5. Small Hairpin RNA (shRNA) Transfection System

A549 cells were seeded in a 10 cm petri dish. After 24 h, cells were transfected with shGFP and shAtg5 by lipofectamine 2000 (Invitrogen, Waltham, MA, USA) and incubated at 37 °C in 5% CO_2_ overnight. The shRNA ATG5 target sequences were #1: CCTTTCATTCAGAAGCTGTTT and #2: CCTGAACAGAATCATCCTTAA. The shRNA GFP target sequence was ACAACAGCCACAACGTCTATA. All shRNAs were purchased from National RNAi Core Facility, Academia Sinica, Taiwan. 

### 2.6. RNA Extraction

Total RNA of samples was extracted by Trizol reagent (MDBio, Inc., Taiwan) and incubated with chloroform (Sigma) at room temperature for 10 min. The mixture was centrifuged at 12,000 rpm for 15 min at 4 °C and the supernatant was transferred to new Eppendorf tubes containing 0.5 mL isopropanol (Merck KGaA, Darmstadt, Germany) and then precipitated at −70 °C overnight. The supernatant was discarded by centrifugation at 12,000 rpm for 8 min at 4 °C. The pellets were washed with 75% ethanol and then centrifuged at 7500 rpm for 5 min at 4 °C; washing and centrifugation were then repeated. The RNA pellets were air-dried for 3 to 5 min and dissolved by DEPC H_2_O.

### 2.7. Reverse Transcription

RNA was transcribed to cDNA by High-Capacity cDNA Reverse Transcription Kits (Thermo Scientific, Waltham, MA, USA). RNA sample (2 µg/10 µL) was mixed with 10 µL 2× RT Master Mix into each tube. The reverse transcription reactions were performed at 37 °C for 2 h in 20 μL of the RT-buffer mixture. In addition, a D8B anti-sense cDNA primer was used to initiate cDNA synthesis [32].

The primer sequence of D8B: CTGCAGAGAACCTUTTGATTC.

### 2.8. Reverse Transcription for DENV Negative-Strand RNA

RNA was transcribed to cDNA by High-Capacity cDNA Reverse Transcription Kits. RNA sample (2 µg/10 µL) was mixed with 10 µL 2× RT Master Mix into each tube. The reverse transcription reactions were performed at 37 °C for 2 h in the presence of Taq-3.2 primer for the negative strand-specific reverse transcription in 20 μL of RT-buffer mixture. [33]. After negative strand-specific reverse transcription, Taq and Taq-3.1 primers were used to amplify the 127 base pair (bp) sequences by PCR [34].

Primer sequences:

Taq-3.2: 5′- CGGTCATGGTGGCGAATAAGCAGATCTCTGATGAATAAC-3′ 

Taq: 5′- CGGTCATGGTGGCGAATAA-3′

Taq-3.1: 5′- TTGTCAACTGTTGCACAGTCG-3′

### 2.9. Polymerase Chain Reaction (PCR)

DENV2 5′-end N1A-E1 (358 bp) and NS1 (258 bp) cDNAs were amplified by PCR using the following primers: 

N1A-E1 Forward: 5′-AUAAUTTUTTAGTCTACGTGGACCGACAAAGAC-3′

N1A-E1 Reverse: CAGATCTCTGATGAATAACC-3′

NS1 Forward: 5′-CACAGATAACGTGCATACATGGAC-3′ 

NS1 Reverse: 5′-TGAGGCCGCAGAGATCG-3′

The conditions of the thermal cycler were programmed as follows. Denaturation was conducted at 94 °C for 5 min. The cDNA generated in the presence of RT-primer was subjected to 35 cycles of DNA amplification with sequential steps at 94 °C for 30 s, 58 °C for 30 s and 72 °C for 1 min. 

### 2.10. Autophagosome Purification

The cells with or without DENV infection were suspended in 0.4 mL 10% sucrose and mixed with 0.5 mL 1 M Hepes/0.1 M EDTA and homogenized using a Dounce homogenizer. This homogenate was diluted with homogenization buffer (HB; 0.25 M sucrose, 10 mM HEPES, 1 mM EDTA, pH 7.3) containing 1.5 mM glycyl-l-phenylalanine 2-naphthylamide and 1% dimethyl sulfoxide (DMSO). After incubation for 7 min at 37 °C to destroy the lysosomes, the homogenate was cooled to 4 °C. The extraction was performed as previously reported [35,36].

### 2.11. Transmission Electron Microscope (TEM)

The cells and autophagosome fraction (AP) were fixed with 2.5% glutaraldehyde in 0.1 M cacodylate buffer for 10 min at 4 °C, followed by three-time treatments of 0.1 M cacodylate buffer containing 4% sucrose for 15 min at 4 °C and post-fixed in 1% osmium tetroxide in 0.1 M cacodylate buffer at RT and washed two times with 0.1 M cacodylate buffer containing 5% sucrose 15 min at RT. The followed by 50%, 70%, 85% and 95% ethanol dehydration for 10 min each and three times with 100% ethanol for 20 min. The sample was treated with propylene oxide two times for 10 min before exposure to propylene oxide/Epikote. After soaking the cells with Epikote two times for 8 h, the cells were embedded with 47.5 μL of Epikote (Glycide ether 100), 24.5 μL of dodecenyl succinic anhydride and 28.5 μL of methylnorbornen-2,3-dicarboxylic anhydride after mixing for 30 min. DMP-30 [2,4,6-tris(dimethylaminomethyl) phenol] (1.5 mL) was then added for 24 h at 60–70 °C. The ultrathin sections of the A549 cell pellet with or without DENV infection and autophagosome fraction (AP) on the nickel grids were treated with 10% H_2_O_2_ for 10 min. After washing with PBS, the grids were treated with protease K (10 µg/mL) at 37 °C for 15 min. Grids were then washed in PBS and blocked for 30 min followed by primary antibody treatment. Grids were incubated with anti-mouse IgG (18 nm Gold) secondary antibody (Abcam, MA, USA) or anti-rabbit IgG (12 nm Gold) secondary antibody (Abcam) for 1 h. The sections on the grids were stained with saturated uranyl acetate and lead citrate and investigated under an electron microscope (HITACHI-7000, Tokyo, Japan) [36]. The microscope voltage is 60 KV; the magnification is 10,000× for cell and 70,000× for purified autophagosome.

### 2.12. Statistical Analysis 

Data are presented as the mean ± SD values (error bars). Differences between the experimental and control groups were analyzed by Student’s *t*-test. The symbol represents *: *p* < 0.05; **: *p* < 0.01 and ***: *p* < 0.001.

## 3. Results

### 3.1. DENV2 Increases LC3-II Level, Autophagic Vesicles and Viral Proteins Are Colocalized with LC3 Protein in Infected A549 Lung Cancer Cells

**Figure 1:**
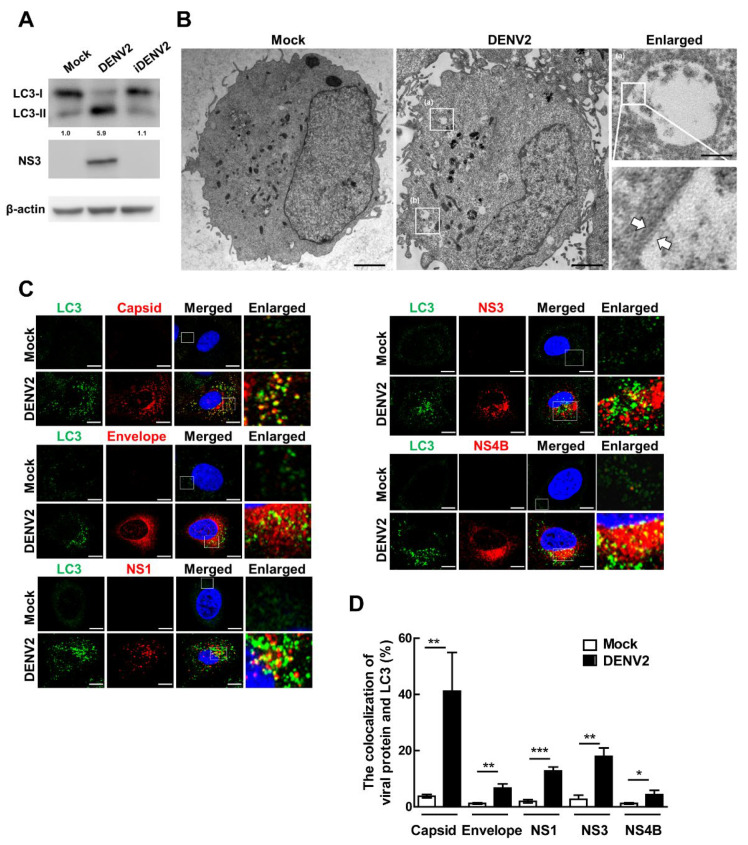
DENV2 increases LC3-II level, autophagic vesicles and viral proteins are colocalized with LC3 protein in infected lung cancer cells. (**A**) A549 cells were treated with mock, live DENV2 (MOI = 10), or heat-inactivated DENV2 (iDENV2; MOI = 10) for 36 h. Protein was extracted from the whole cell lysate and analyzed by immunoblotting for NS3 and LC3 expression. β-actin was used as the internal control. (**B**) A549 cells after infection were fixed at 36 h post-infection (p.i.) and observed under TEM. Scale = 2 µm. The white arrow represents double-membrane autophagosome in (a). Single membrane autophagic vesicles in (b). Scale bar = 0.2 µm in the enlarged picture (a). (**C**) Cells were infected with or without DENV2 (MOI = 10) for 36 h and examined for the colocalization of LC3 (green) and DENV2-Capsid, Envelope, NS1, NS3 and NS4B (red) under confocal microscopy. The yellow dots indicate colocalization of DENV2 proteins and LC3. Scale bar = 10 µm. (**D**) Quantification of the colocalization between DENV2 proteins (capsid, envelope, NS1, NS3 and NS4B) and LC3 puncta in DENV2-infected cells. The percentage of colocalization was quantified by counting the number of yellow dots in the cells and the cells containing more than five yellow dots represent colocalization-positive. Three fields of 10 cells each were countered under each experimental condition. The data shows Mean ± SD and the *p* values were determined by Student’s *t*-test analysis. * *p* < 0.05, ** *p* < 0.01, *** *p* < 0.001.

We previously reported that DENV induces autophagy in liver cancer Huh7 cells and plays a promoting role in viral replication [22,37]. Here, we used live and heat-inactivated DENV2 to infect lung cancer cells A549 [31] and demonstrated that the conversion of the autophagy marker LC3-II protein increased, which was accompanied by a higher level of DENV2 NS3 protein in the DENV2 infection cells compared to the non-infection (Mock) and heat-inactivated DENV (iDENV2) cells (Figure 1A). The quantification of LC3 conversion (LC3-II/LC3-I) is widely used to evaluate the level of autophagic activity. Furthermore, more autophagic vesicles including double-membrane autophagosome and single-membrane autophagic vesicles were detected in DENV2-infected A549 cells compared to the Mock infected cells under transmission electron microscopy (TEM) (Figure 1B).

As a defense response, autophagosomes selectively engulf pathogens for degradation in the host cells. In this study, we were interested in identifying the DENV2 components that are associated with autophagosomes in infected cells. We found that the number of fluorescent green LC3 punctate structures (representing autophagic vesicles) was increased after DENV2 infection by confocal microscopy. The infected cells were examined to determine the presence or absence of DENV2 structural proteins capsid (C) and envelope (E) and non-structural proteins (NS1, NS3 and NS4B) as well as their colocalization with autophagy LC3 protein. We detected these five proteins (red) and showed varying degrees of colocalization with LC3 (green) in infected cells, with capsid protein showing the greatest colocalization, followed by NS3, NS1, envelope and NS4B protein (Figure 1C,D).

### 3.2. DENV2 Capsid, Envelope, NS3, NS4B and Cellular HMGB1 Proteins Were Abundantly Detected in the Purified Autophagosomes of Infected Lung Cancer Cells

**Figure 2:**
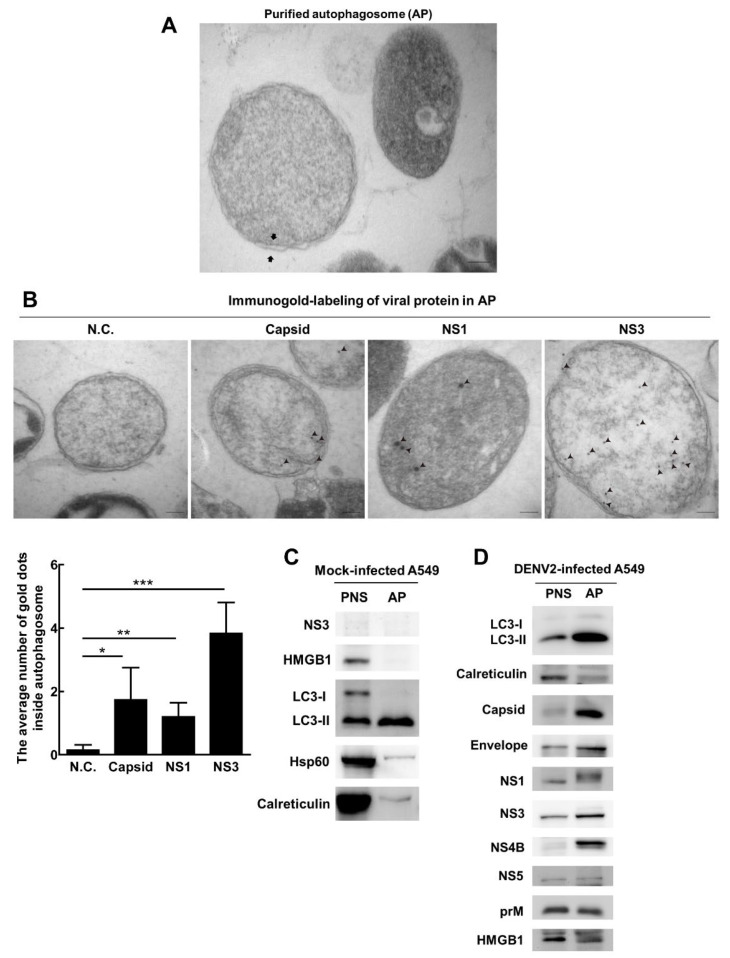
DENV2 capsid, envelope, NS1, NS3 and NS4B proteins are abundantly detected in the purified autophagosomes of infected lung cancer A549 cells. (**A**) Cells were infected with DENV2 (MOI = 10) for 36 h followed by treatment with chloroquine (CQ, 50 μM) for another 24 h to block lysosome fusion and degradation. The autophagosome was purified by sucrose gradient centrifugation and observed under TEM. Scale bar = 100 nm. The arrow indicates the double-membrane of the purified autophagosome. (**B**) The protein expression of the capsid, NS1 and NS3 was detected by specific antibodies. Negative control (N.C.) used IgG antibody for immunogold-labeling. Immuno-gold labeled NS1 (18 nm gold beads), Capsid (12 nm gold beads) and NS3 (12 nm gold beads) in the purified autophagosomes were detected under the TEM. The quantification of gold particles for each protein inside autophagosomes was shown. Scale bar = 100 nm. * *p* < 0.05, ** *p* < 0.01, *** *p* < 0.001. (**C**) Lung cancer cells (A549) without DENV infection and culture for 36 h followed by blocking autophagosome and lysosome fusion with chloroquine (CQ, 50 μM) for another 24 h. Sucrose gradient centrifugation was conducted to obtain purified AP and PNS. A total of 10 μg protein from AP and PNS was loaded in SDS PAGE and analyzed by electrophoresis. The protein expression of LC3, NS3, HMGB1, Hsp 60 and Calreticulin was determined by immunoblotting. (**D**) Lung cancer cells (A549) were infected with DENV2 (MOI = 10) for 36 h followed by blocking autophagosome and lysosome fusion with CQ (50 μM) for another 24 h. Sucrose gradient centrifugation was conducted to obtain purified autophagosome (AP) and post-nucleus supernatant (PNS). A total of 10 μg protein from AP and PNS was loaded in SDS PAGE and analyzed by electrophoresis. The protein expression of LC3, LAMP1, capsid, envelope, NS1, NS3, NS4B, NS5, prM and HMGB1 was determined by immunoblotting using specific antibodies. LAMP1 is the marker of the lysosome and calreticulin is the marker of the endoplasmic reticulum.

To clarify whether DENV2 proteins are associated with the autophagosomes in infected cells, the autophagosomes of DENV2-infected A549 cells were purified by density gradient centrifugation. Double-membrane autophagosomes were observed in the purified autophagosome fraction under TEM (Figure 2A). DENV2 capsid, NS1 and NS3 proteins showing higher colocalization with LC3 (Figure 1D) were confirmed in the purified autophagosomes under TEM using immunogold-labeled antibodies (Figure 2B, arrowhead). Most importantly, no virus-like particles were found in the purified autophagosomes.

We purified the autophagosome (AP) from A549 cells without DENV infection and showed very low levels of ER (calreticulin) and mitochondria (Hsp60) contamination (Figure 2C). Parallelly, in cells with DENV infection, the level of the lysosome (LAMP1) and ER (calreticulin) in AP is low. These results represent successful autophagosome purification, which was confirmed by the abundant amount of LC3-II (autophagosome marker) in the AP fraction compared to the post-nucleus supernatant (PNS) by immunoblotting (Figure 2D). Similarly, high amounts of DENV2 capsid, envelope, NS1, NS3 and NS4B proteins, accompanied with equal amounts of DENV2 prM and NS5 protein, as well as host high mobility group box 1 (HMGB1) proteins, were detected in the purified autophagosomes (Figure 2D). A similar result was seen in DENV2-infected Huh7 cells (Supplemental Figure S1). Altogether, our results demonstrated that DENV2-related proteins and host cell HMGB1 proteins, but not viral particles were present with the autophagosome in two infected cell lines.

### 3.3. DENV2 Capsid, Envelope, NS3 and NS4B Proteins Were Not Degraded by the Autophagic Degradation Process 

**Figure 3:**
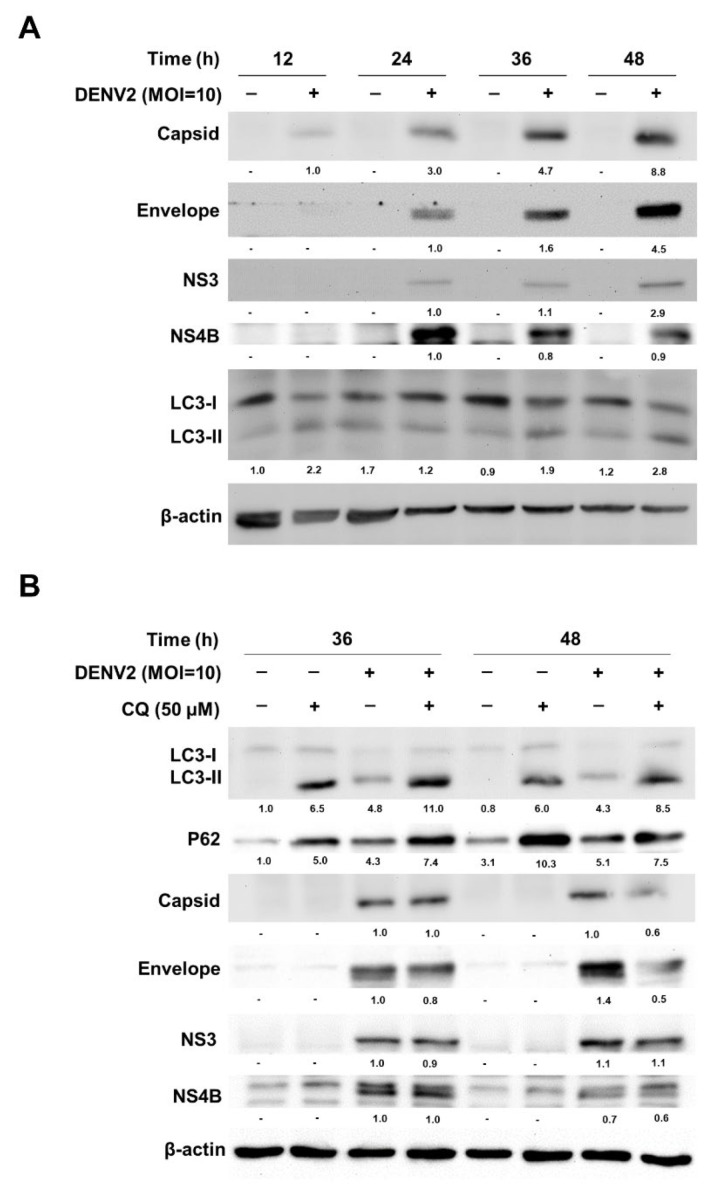
DENV2 proteins were not degraded by the conventional autophagic degradation process. (**A**) The protein expression levels of DENV2 capsid, envelope, NS3 and NS4B, as well as autophagy LC3 at 12, 24, 36 and 48 h in A549 cells with or without DENV2 (MOI = 10) infection, were investigated by immunoblotting using specific antibodies. (**B**) A549 cells were infected with or without DENV2 (MOI = 10) for 12 and 24 h followed by co-treatment with CQ (50 μM) for another 24 h. The whole-cell lysate extracted from each group was analyzed for the expression levels of DENV2 capsid, envelope, NS3 and NS4B, as well as autophagy LC3 and p62 proteins by immunoblotting using specific antibodies. β-actin was used as the internal control.

As a part of the immune system, autophagy is responsible for the recruitment and degradation of harmful molecules, damaged organelles and pathogens [38]. Here, we conducted a time-course investigation of the levels of DENV2 capsid, envelope, NS3 and NS4B proteins from 0 h to 48 h p.i. during a 12 h interval. Our data showed that the levels of DENV2 capsid, envelope, NS3 and NS4B proteins each gradually increased with the duration of infection and the kinetics of these protein expressions was similar to that of LC3-II expression (Figure 3A). Blocking the autophagic degradation with lysosome, infected cells were treated with CQ and the results showed that the accumulation of LC3-II and p62 proteins increased in the CQ group compared to the untreated group, indicating that CQ effectively blocked the autophagic degradation machinery (Figure 3B). The levels of DENV2 capsid, envelope, NS3 and NS4B proteins did not accumulate in the CQ treatment group compared to the non-treatment group in DENV2-infected cells (Figure 3B), indicating that DENV2 capsid, envelope, NS3 and NS4B proteins were not affected by autophagic degradation machinery.

### 3.4. DENV2 RNAs Are Identified in Autophagosomes and the Purified Autophagosomes Are Infectious

We and other groups have reported that DENV infection induces autophagosome formation, which promotes DENV replication either by using the autophagosome double membrane as part of the replication complex or by degradation of lipid droplets to provide ATP energy [22,39]. However, whether the autophagosome selectively recruits and conveys the viral components, including the viral genome, remains poorly understood. 

**Figure 4:**
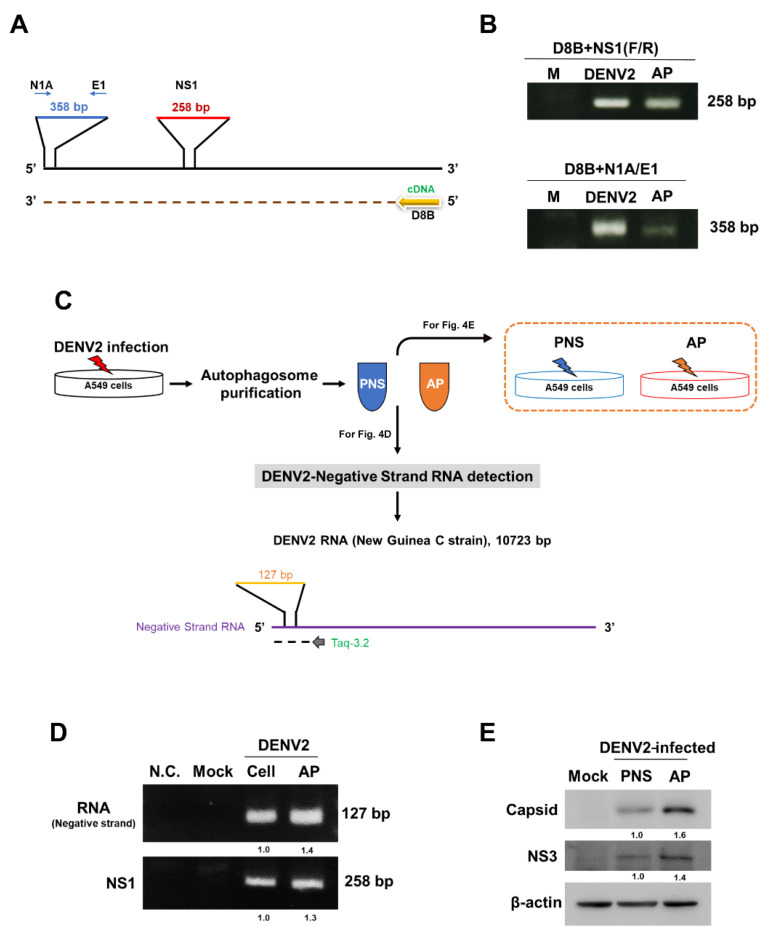
DENV2 RNAs are identified in the purified autophagosomes, which are infectious. (**A**) Primer D8B at the 3′end of the DENV genome was used for the initiation of cDNA synthesis. The PCR primers N1A-E1 and NS1 were used for PCR amplification and the corresponding fragments and sizes were 258 and 358 bps, respectively. (**B**) RNA was extracted from A549 cells with (DENV) or without DENV2 infection (Mock) and purified autophagosomes from infected cells (AP). The expression of the 5′end N1A-E1 (358 bp) and middle NS1 (258 bp) region of the DENV2 genome was measured by RT-PCR. (**C**) The experimental design for (**D**,**E**). The autophagosome was purified from DENV2-infected A549 cells. The upper panel shows the purified autophagosome and PNS were treated with other uninfected A549 cells. The lower panel shows the primer region in the DENV2 genome for cDNA synthesis and PCR of negative-strand DENV2 RNA. (**D**) RNA was extracted from A549 cells with DENV2 (Cell) or without DENV2 infection (Mock) and purified autophagosomes from infected cells (AP). The expression of negative-strand RNA and NS1 of DENV2 was determined by RT-PCR. N.C.: control of RT-PCR (without cDNA template). (**E**) A549 cells were cultured with purified autophagosome (AP) or PNS fraction for 72 h, followed by measuring the levels of DENV2 capsid and NS3 proteins by immunoblotting using specific antibodies. N.C.: control of RT-PCR (without cDNA template). The data shows Mean ± SD and the *p* values were determined by Student’s *t*-test analysis.

The use of full-length genome RNA to generate infectious viruses was first reported by Racaniello and Baltimore (1981), who synthesized infectious type 1 poliovirus from the plasmid cDNA [40]. We previously revealed an in vivo approach to detect and produce matured DENVs with infectivity from the full-length genome RNA reverse-transcribed from the plasmid cDNA [30]. Moreover, we previously reported a RT-PCR method to identify DENV full-length positive-strand RNA genome in infected cells [32]. Briefly, we used a primer (D8B), which recognizes the 3′-end of the +RNA genome, to reverse-transcribe the RNA genome to cDNA (Figure 4A). To confirm the synthesis of the full-length RNA genome, we applied PCR to amplify the 3′-end of the cDNA sequence (5′end of RNA sequence) using a N1A/E1 primer set and we also used the NS1 region primer set to amplify the cDNA sequence in the middle region (Figure 4A). Our RT-PCR amplified larger amounts of a 258 bp fragment (5′-end of the genome) and a 358 bp of NS1 fragment (middle) in the purified autophagosome (AP) and the DENV2-infected cells (DENV2, as a positive control), compared to no amplification in mock infection (M) (Figure 4B), indicating that the purified autophagosomes indeed contained the full-length DENV RNA genome. Moreover, we also detected DENV2 negative-strand RNA, which was de novo synthesized to form a replication complex during viral replication (Figure 4C diagram and 4D, 127 bp fragment) accompanied with DENV-NS1 RNA expression (Figure 4D, 258 bp) in the purified autophagosome by RT-PCR. These data indicate that besides DENV proteins, the autophagosome possibly recruits both viral genome and replicative-form RNA of DENV. Because the viral particles were not identified in the purified autophagosome and importantly the replicative full-length viral +RNA was identified in the purified autophagosome, suggesting that DENV-induced autophagosome functions not only as the docking site and ATP producer for replication but also as the vesicles to convey infectious DENV components.

Furthermore, we co-cultured either the purified autophagosomes (AP) or the post-nucleus supernatant (PNS) from DENV2-infected cells with A549 cells (Figure 4E) to clarify whether the purified autophagosomes are infectious. Notably, higher levels of DENV2 capsid, as well as NS3 proteins, were detected in the AP fraction compared to the PNS fraction and no viral proteins were detected in the Mock treatment by immunoblotting (Figure 4E), indicating that the purified autophagosomes contained much more infectious materials. This finding is consistent with the result displayed in Figure 4B indicating that DENV-associated autophagosomes are infectious.

### 3.5. DENV2 Triggers HMGB1 Exocytosis Partially through Unconventional Secretory Autophagy

**Figure 5:**
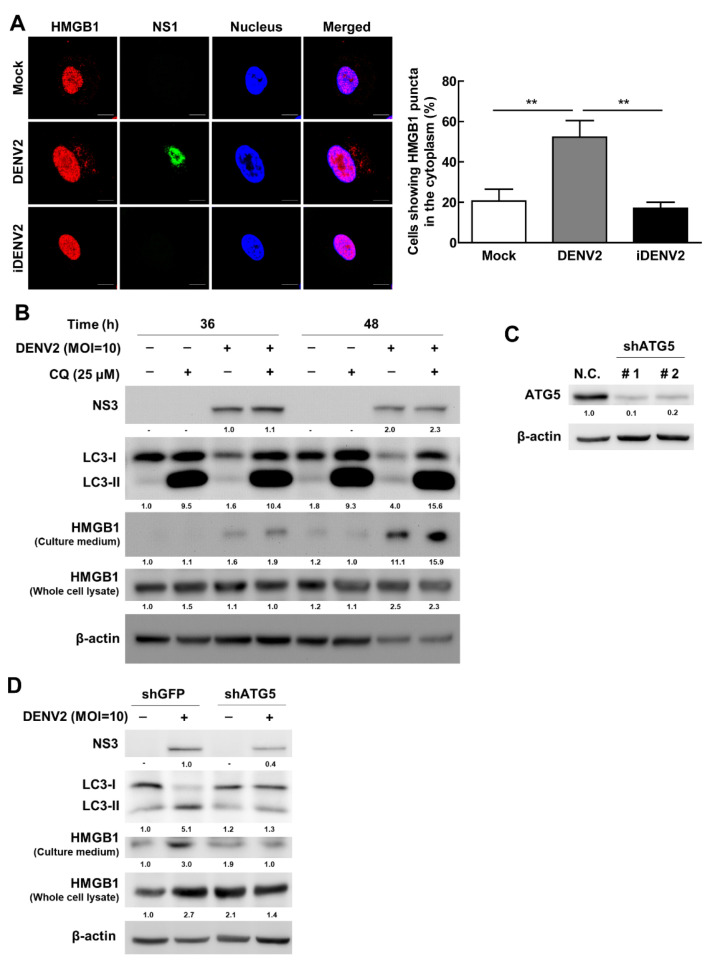
DENV2 triggers HMGB1 exocytosis through unconventional secretory autophagy. (**A**) A549 cells were infected by DENV2 (MOI = 10), or heat-inactivated DENV2 (iDENV2; MOI = 10) for 36 h. The mock treatment was negative control without infection of DENV. Cells were examined for the localization of DENV-NS1 (green) and HMGB1 (red) by confocal microscopy. The quantification was the percentage of cells showing HMBGB1 puncta in the cytoplasm. Scale bar = 10 µm. ** *p* < 0.01. (**B**) A549 cells were infected with or without DENV2 (MOI = 10) for 12 h and 24 h followed by co-treatment with CQ (25 μM) for another 24 h. The whole-cell lysate and extracellular supernatant were analyzed by immunoblotting for LC3, NS3 and HMGB1 expression using specific antibodies. (**C**) The levels of ATG5 expression in negative control (shGFP) cells or two shATG5 clones were evaluated by immunoblotting. (**D**) The levels of cellular ATG5, LC3, HMGB1, and DENV-NS3 in shGFP or shATG5 cells and released-HMGB1 expression in culture medium with or without DENV2 infection were investigated by immunoblotting.

HMGB1 is a nuclear DNA-binding protein, which loosely binds to chromatin and is present in almost all eukaryotic cells. Extracellular HMGB1 functions as a damage-associated molecular pattern (DAMP), which has potent proinflammatory effects in many inflammatory diseases. Kim et al. reported that heat shock protein 90 (HSP90AA1) mediates the translocation of HMGB1 from nuclei to the cytoplasm and secretory autophagy is accompanied by the multivesicular body (MVB) formation-mediated HMGB1 secretion [41]. In this study, we found an increased number and translocation of the HMGB1 puncta formation in the cytoplasm in DENV2-infected A549 cells (Figure 5A), indicating that DENV2 infection increased HMGB1 the translocation from the nucleus to the cytoplasm and to be puncta formation. Furthermore, our data showed that HMGB1 was detected in the purified autophagosomes from DENV2-infected A549 and Huh7 cells (Figure 2D and Supplemental Figure S1B). These data indicate that HMGB1 associated with the autophagosome in DENV2-infected cells. To clarify whether autophagosome-associated HMGB1 was degraded by the autophagic degradation system, chloroquine (CQ) was used to block lysosome fusion and degradation. At 24 h post-infection (p.i.), CQ was added to the infected cells for another 24 h. Our data showed that LC3-II protein accumulated in the CQ treatment group, indicating that the autophagic flux was effectively blocked. Under these conditions, the HMGB1 expression level of whole cell lysate did not accumulate (Figure 5B), implying that HMGB1 was not degraded by the autophagic degradation machinery. We further investigated whether autophagosomes play a role in HMGB1 release after DENV2 infection by detecting the expression of HMGB1 in the extracellular environment at 36 h and 48 h p.i. We determined that HMGB1 exocytosis (HMGB1 expression level in culture medium) was increased in a time-dependent manner (Figure 5B). We then silenced ATG5 expression by short hairpin RNA (shATG5) to suppress the autophagic process in A549 cells. The ATG5 proteins were downregulated by RNA interference in A549 cells (Figure 5C). The ATG5 knockdown cells were infected with DENV2 at the MOI of 10 for 36 h. The expression of LC3-II protein was significantly decreased in DENV-infected ATG5 knockdown cells and the level of HMGB1 in the culture medium was decreased in shATG5 cells compared to the control shGFP cells. In contrast, the intracellular level of HMGB1 was not significantly affected (Figure 5D, whole-cell lysate). Moreover, to clarify the role of DENV-induced cell death in HMGB1 release, lactate dehydrogenase (LDH, a marker of cellular cytotoxicity and cytolysis) assay was conducted. We reveal that in DENV infected cells, LDH level was increased, however, the level of LDH showed no significant difference between DENV2-infected shATG5 cells and DENV2-infected control shGFP cells (Supplemental Figure S2). Therefore, decreased HMGB1 secretion in ATG5 KD cells may result from blockage of the secretory process or suppression of viral replication (Figure 5D). Taken together, our data imply that secretory autophagy also participates in HMGB1 exocytosis in addition to cytolysis of the DENV2-infected A549 cells. Since HMGB1 together with DENV proteins were detected in the autophagic vesicles, it is possible that these autophagic vesicles may utilize a similar trafficking pathway like HMGB1 to exocytosis of the autophagic vesicles harboring infectious DENV components such as full-length RNA genome. 

## 4. Discussion

**Figure 6:**
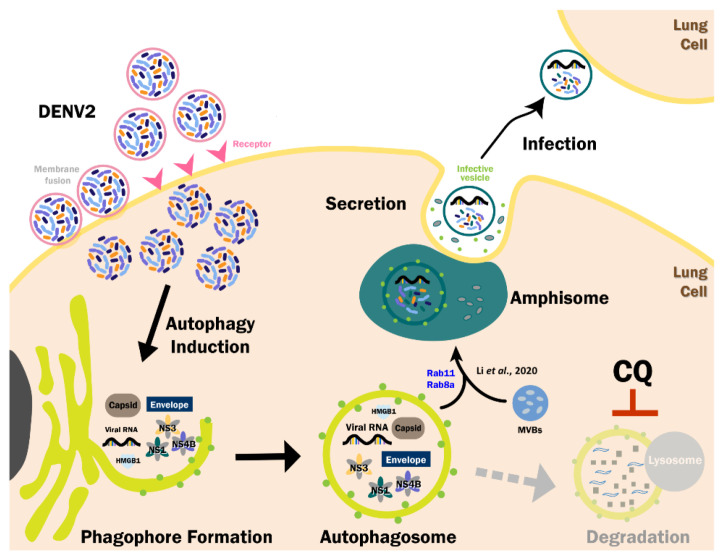
A schematic hypothesis of the role of autophagy in DENV infection and exocytosis. DENV2 triggers the endoplasmic reticulum to form the isolation membrane in the presence of multiple factors, followed by progression to form the mature autophagosome, which may selectively recruit DENV2 proteins and RNA, including negative-stranded and full-length DENV RNAs. These infectious autophagic vesicles may be released from the infected cells partially through the secretory autophagy pathway.

We and other groups have reported that DENV infection induces autophagosome formation, which may serve as the docking site for virus replication [22,42]. Heaton et al. (2010) revealed that DENV-induced lipophagy regulates lipid metabolism by selectively recruiting lipid droplets and triglycerides to release free fatty acids followed by increasing cellular β-oxidation to generate ATP. These processes were shown to be required for efficient DENV replication [39]. DENV-NS4A proteins have been found to play an essential role in autophagy induction [25]. However, the relationships among autophagy, DENV proteins, genome RNA, viral particles and viral replication remain poorly understood. In this study, we found that part of the DENV proteins and genomic RNAs were selectively associated with the autophagosome during viral infection. Notably, purified autophagosomes containing specific DENV2 proteins and full-length genomic RNA were found to be infectious (Figure 6). Although the viral particles were not observed in the purified autophagosome under TEM, full-length positive genome RNA can de novo generate viral particles both in vitro and in vivo. Our data were providing compelling evidence that the autophagic vesicles, both intracellular and extracellular, are infectious. Our findings support the results of Li et al. (2020) who identified a Lyn-dependent exit route of DENV-containing secretory organelles, which are infectious and might affect tissue tropism [16].

In our study, DENV increased LC3-II levels at 36 h and 48 h p.i. (Figure 3 and Figure 5B) may be caused by increased autophagic activity or blockage of autophagic degradation, which can be clarified by autophagic degradation inhibitors such chloroquine (CQ) or bafilomycin A1. It is known that DENV2 internalization is mediated by clathrin-mediated endocytosis followed by trafficking to an endosomal compartment with a low-pH environment, which is required for entry, uncoating of viral particles and promoting the deposition of viral RNA into the host cytoplasm [43,44]. Bafilomycin A1 blocks degradation by preventing the acidic environment of the organelles. Differently, CQ inhibits autophagic degradation by blockage of the fusion of autophagosome and lysosome without affecting pH [45]. To avoid affecting the viral invasion process and viral replication, we selected CQ to clarify the mechanism of DENV-induced autophagy. Our data showed that CQ effectively blocked autophagic degradation in uninfected A549 cells demonstrated by significant accumulation of LC3-II and p62 proteins (Figure 3 and Figure 5). However, in DENV2 infected cells, LC3-II, as well as p62 accumulation, was not further increased either at 36 h or 48 h p.i. For example, in Figure 3B at 36 h p.i., the accumulation of LC3-II (11.0 in lane 4) after subtraction of the basal accumulation of 6.5 in lane 2 is 4.5, which is smaller than 4.8 in lane 3. Similar results were seen in Figure 3B at 48 h and Figure 5B at 36 h and 48 h p.i.. These data imply that DENV2 infection mainly leads to blockage of autophagic degradation in A549 cells. Similarly, Meza et al. (2015) reported that DENV infection of Huh7 cells induced an initial activation of autophagic flux followed by inhibition of general and specific autophagy at 36 h p.i. [46]. However, in A549 cells, whether DENV could induce an earlier autophagic flux remains to be determined.

To the best of our knowledge, we are the first to successfully purify autophagosomes from DENV2-infected A549 and Huh7 cells and to reveal that DENV2 proteins and genome RNAs existed in these autophagosomes. The numbers of identified peptide sequences (peptide spectrum matches (PSMs)) of the DENV proteins in the purified autophagosomes are shown in Supplemental Figure S3. All of the DENV structural and nonstructural proteins were detected but in different proportions, indicating that mature viral particles may exist in the autophagosome. We attempted to identify the progeny DENV particles in the purified autophagosomes. However, no typical DENV virion could be observed in the purified autophagosomes under TEM. Similarly, Li et al. (2020) utilized various techniques, including discontinuous sucrose gradient centrifugation and biochemical analysis, to claim that the secretory autophagosome-like vesicles contained mature DENV particles; but the authors did not prove this result under TEM (with the exception of Zika virus) [16]. Our observation implies that the autophagosome may not directly carry the DENV2 virion out of the cell. Instead, it is probable that the infectivity of the intracellular autophagosome or the extracellular autophagosome-like vesicle originates from the full-length DENV RNA. Positive full-length viral RNA can de novo generate infectious virus when entering neighboring uninfected cells through endocytosis [30]. This speculation warrants further investigation.

Previous studies revealed that HMGB1 is released passively by DENV-infected non-immune cells. However, in the present study, we discovered that the level of HMGB1 in the culture medium was reduced in ATG5 knockdown A549 cells compared with control knockdown cells (Figure 5D). Moreover, the level of LDH showed no significant difference between DENV2-infected ATG5 knockdown cells and DENV2-infected control cells (Supplemental Figure S2). Taken together, these data imply that secretory autophagy plays a partial role in HMGB1 release in DENV2-infected A549 cells. Similarly, secretory autophagy accompanied by the multivesicular body (MVB) formation-mediated HMGB1 secretion has been reported [41]. Here, we detected HMGB1 and DENV proteins, as well as RNAs in DENV-related autophagosomes (Figure 2D and Figure 4B,C,E), which provides strong evidence for our speculation that secretory autophagy participates in the exocytosis of DENV proteins and RNAs.

Moreover, the release of HMGB1 was increased after CQ treatment (Figure 5B) which was similar to other group reported that secretory autophagy may be promoted by inhibiting lysosome degradation [16]. This phenomenon has also been reported in Parkinson’s disease, indicating that the inhibition of degradative autophagic flux induces α-synuclein release through secretory autophagy. The precise mechanism by which lysosome inhibitor triggers the secretory autophagy pathway remains unclear and, therefore, further research is needed.

DENV infection triggered exocytosis of autophagy-related vesicles to infect neighboring cells and evade immune system surveillance. These autophagic vesicles contain DENV NS1, prM, envelope proteins and RNA [15]. Herein, we further confirmed the presence of DENV2 RNA, capsid, envelope, NS1, NS3 and NS4B proteins in the purified autophagosome of infected lung and liver cells (Figure 2D and Supplemental Figure S1). We also reveal that DENV2-induced autophagosomes were infectious, possibly through full-length DENV2 genome RNA (Figure 4E). This finding is consistent with a report by Li et al. (2020) showing that secretory autophagy mediated exocytosis of LC3+ vesicles containing infectious viruses. In summary, our results indicate that extracellular infectious dengue vesicles may originate from autophagosomes containing infectious DENV RNA.

Following the maturation of the double-membrane autophagosome, it proceeds to fuse with multivesicular bodies (MVBs). In the early endosome, the cargoes within are either sorted to the vesicles that proceed to late endosomes/MVB or recycle back to the plasma membrane [47]. The late endosomes/MVB fuse with lysosomes in which their cargo is degraded and their degraded products such as amino acids are made available for reuse by the cell. MVBs and lysosomes transport cargos from the endocytic and autophagy pathways [48]. Both degradative and secretory autophagosomes are known to fuse with MVBs to generate amphisomes before fusion with lysosome or plasma membrane, respectively [27]. Degradative and secretory autophagosomes undergo interconversion or redirection of the substrate to process whether the substrate can be secreted or degraded. Given the critical role of MVB biogenesis, they regulate the interplay between autophagy and protein secretion. Exosomes are generated from the fusion of MVB with the plasma membrane and release their accumulated small vesicles with a size of 40–100 nm into the extracellular matrix [49]. A recent study has observed an analogous process in maturing reticulocytes in which LC3-positive MVB were shown to fuse with the plasma membrane in vitro and similar vesicles were identified in peripheral blood [16].

Approximately 70 different Rab proteins in humans are involved in vesicle trafficking and play key roles in the regulation of the autophagic process. Rab5 is a regulator of the early steps of autophagosome formation [50]. Rab7 is required for the fusion of the autophagosome with late endosomes or lysosomes [51,52]. Rab8a and Rab8b control the autophagy secretion and degradation process, respectively [29]. Rab proteins regulate vesicle trafficking and autophagic processes are important for the DENV life cycle. Rab8 was colocalized with DENV2 in HpeG2 cells and viral entry, release and production were significantly reduced in Rab8-mutated cells [53]. Rab18 participates in the interaction of fatty acid synthase (FASN) with DENV NS3 and the recruitment of the FASN-NS3 complex to the sites of viral replication/assembly, including ER and LDs, during DENV2 infection [54]. Furthermore, Rab5 is essential for DENV entry [55]. Li et al. (2020) reported that Rab11, the transferrin receptor and LC3 were enriched in membranes of both recycling endosomes, as well as exosomal and secretory autophagic vesicles [16]. In this study, fifty Rab proteins were detected and overexpressed in the purified autophagosomes from DENV2-infected A549 cells which compare to uninfected cells by proteomic analysis, indicating that Rab proteins may participate in autophagic vesicle trafficking harboring DENV proteins and RNA (Supplemental Table S1). Rab8a and Rab11 were detected in the purified autophagosomes of DENV-infected cells, which further supports the role of secretory autophagy in DENV2-infected cells. Furthermore, SNARE complex proteins (for example syntaxin 3, syntaxin 4, SNAP29 and Sec22b) were also detected in the purified autophagosomes from DENV2-infected A549 cells by proteomic analysis, indicating that Rab and SNARE proteins may participate in autophagic vesicle trafficking harboring DENV proteins and RNA. However, the identification of the specific SNARE proteins that involve in the fusion of secretory autophagosomes with the plasma membrane needs further study.

In conclusion, the intracellular autophagosomes harboring DENV2 proteins and genome RNA are infectious, however, whether they proceed to extracellular autophagic vesicles with infectivity warrants further exploration.

## Data Availability

Data sharing is not applicable to this article.

## Supplementary Materials

The following are available online at https://www.mdpi.com/article/10.3390/v13102034/s1, Figure S1: DENV2 capsid, envelope, NS1, NS3, and NS4B proteins are detected in the purified autophagosomes of infected liver Huh7 cells but not in uninfected cell, Figure S2: The kinetics of LDH release in DENV-infected cells, Figure S3: The proteomic analysis of DENV2 proteins in the purified autophagosomes from infected A549 cells, Table S1: RAB proteins participate in autophagy and DENV replication. References [56,57,58,59,60,61,62,63,64,65,66,67,68,69,70,71] are cited in the Supplementary Materials.
