# Supplementary material for: The Autophagosomes Containing Dengue Virus Proteins and Full-Length Genomic RNA Are Infectious
Source: Viruses. 2021 Oct 9;13(10):2034. doi: 10.3390/v13102034 (PMC8540618; doi:10.3390/v13102034)
Supplement: Supplementary file 1 [file viruses-13-02034-s001.zip › viruses-1367091-supplementary.pdf]

**Supplemental Materials and Methods**

***Proteomics analysis of autophagosome***

The protein was extracted from the purified autophagosomes of DENV2-infected A549 cells. Proteins were digested by trypsin at 37 °C for 16 h. The peptide fractions were analyzed on a nanoLC-Q ExactiveTM HF mass spectrometer (Thermo Fisher, San Jose, USA). The MASCOT search engine (version 2.5, Matrix Science, MA, USA) was used for protein identification.

**Supplemental Table S1**. RAB proteins participate in autophagy and DENV replication

| RABs | Role in autophagy | Role in DENV |
|---|---|---|
| **RAB1** | Phagophore assembly by regulating ATG9 localization [1,2] Regulation of TORC1 activity [3] | |
| **RAB4** | Autophagosome formation [4] | |
| **RAB5** | Regulation of PIK3C3-BECN1 complex [5] Regulation of TORC1 activity [3] | Virus entry [6] |
| **RAB7** | Microtubular transport of autophagosomes [7] Autophagosome maturation [8] Autophagic lysosome reformation [9] Autophagosome formation [10] Regulation of TORC1 activity [11] | |
| **RAB8** | utophagy-based secretion [12] Autophagosome maturation during antimicrobial autophagy [13] | Virus entry Virus release Infectious virus production [14] |
| **RAB9** | Autophagosome maturation during antimicrobial autophagy [15] Autophagosome formation during ATG5- and ATG7-independent noncanonical autophagy [16] | |
| **RAB11** | Providing ER-derived membrane source for autophagy [17] Maturation of autophagosomes [18] Regulation of TORC1 activity [3] | |
| **RAB18** | Unknown | Membrane trafficking of FASN and NS3 to replication site [19] |
| **RAB24** | Colocalization with LC3 upon autophagy induction [20] | |
| **RAB32** | Required for autophagosome formation [21] | |
| **RAB33** | Autophagosome formation and maturation [22] | |

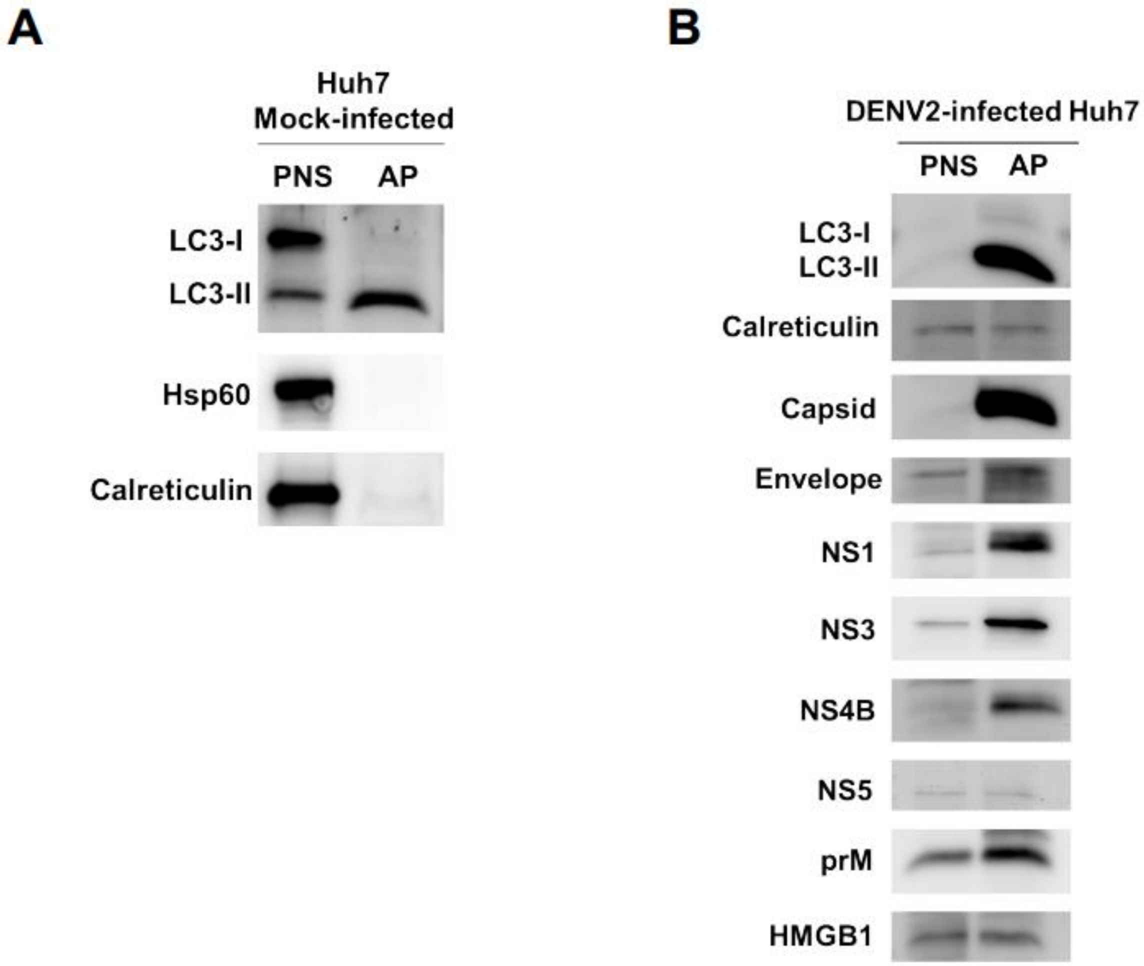


**Supplemental Figure S1**. DENV2 capsid, envelope, NS1, NS3, and NS4B proteins are detected in the purified autophagosomes of infected liver Huh7 cells but not in uninfected cell (**A**) Liver cancer cells (Huh7) without DENV infection and culture for 36 h followed by blocking autophagosome and lysosome fusion with chloroquine (CQ, 50 μM) for another 24 h. Sucrose gradient centrifugation was conducted to obtain purified AP and PNS. A total of 10 μg protein from AP and PNS was loaded in SDS PAGE and analyzed by electrophoresis. The protein ex-pression of LC3, Hsp 60 and Calreticulin was determined by immunoblotting. (**B**) Liver cancer Huh7 cells were infected with DENV2 (MOI=10) for 36 h followed by blocking autophagosome and lysosome fusion with chloroquine (CQ, 50 μM) for another 24 h. Sucrose gradient centrifugation was conducted to obtain purified autophagosome (AP) and post-nucleus supernatant (PNS). A total of 10 μg protein from AP and PNS was loaded in SDS PAGE and analyzed by electrophoresis. The protein expression of LC3, capsid, envelope, NS1, NS3, NS4B, NS5, prM, and HMGB1 was determined by immunoblotting using specific antibodies. Calreticulin in the marker of the endoplasmic reticulum.

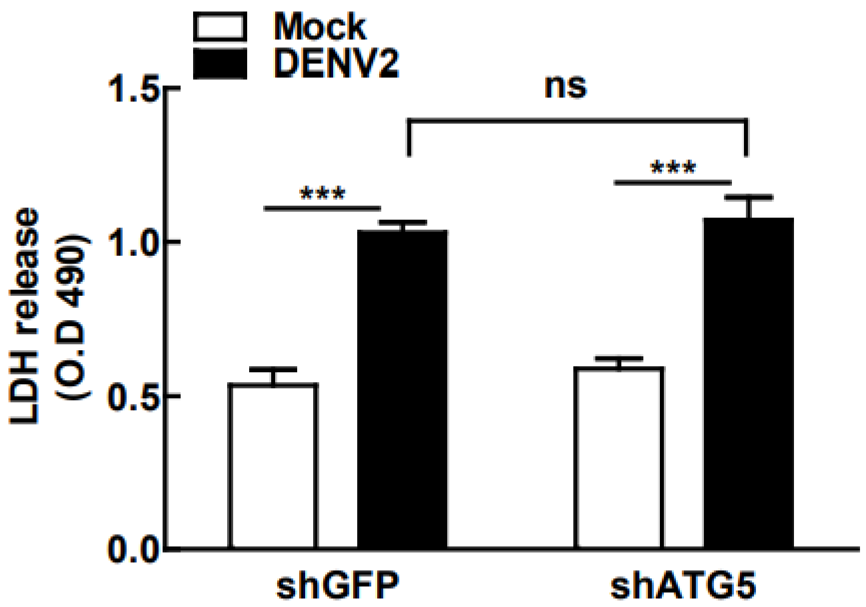


**Supplemental Figure S2**. The kinetics of LDH release in DENV-infected cells The kinetics of LDH release. LDH leakage into the culture medium was measured with or without DENV2 infection in shGFP or shATG5 cells at 36 h. Using LDH Assay Kit / Lactate Dehydrogenase Assay Kit (ab102526) and evaluation of LDH activity released into the media. The data represent O.D. value from three independent experiments. ns: not significant.

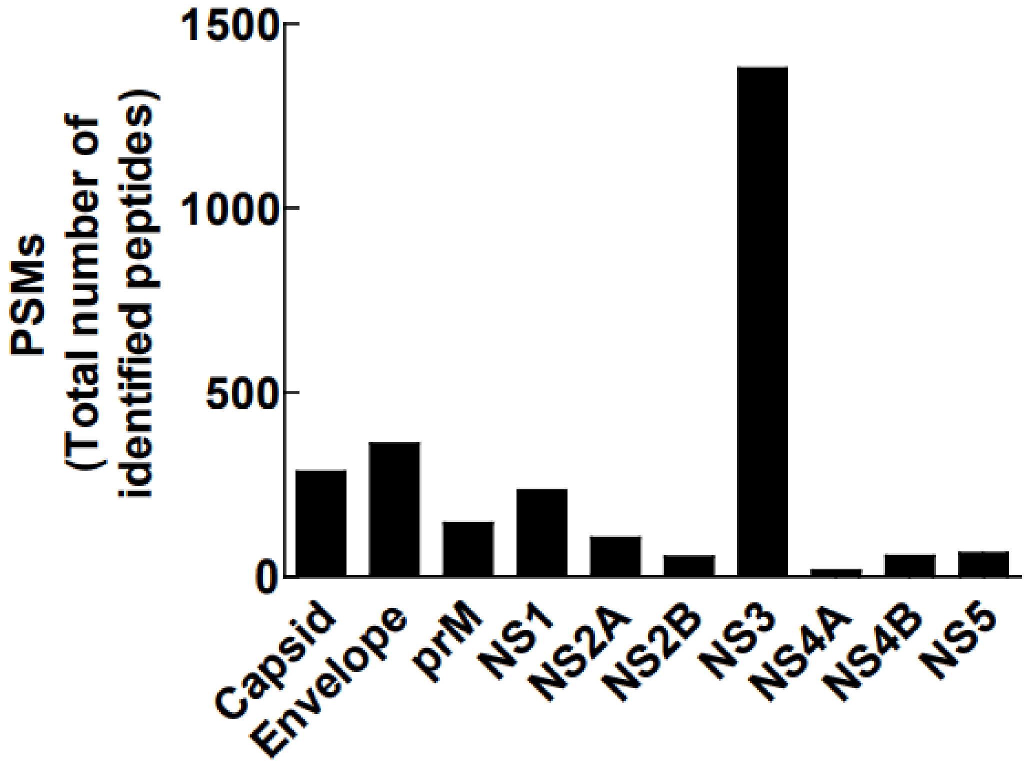


**Supplemental Figure S3.** The proteomic analysis of DENV2 proteins in the purified autophagosomes from infected A549 cells Proteins extracted from purified autophagosome in DENV2-infected A549 cells were analyzed by LC/MS/MS quantitative proteomics. The total number of identified peptide sequences of DENV2 proteins was shown as peptide spectrum matches (PSMs).

## References


1. Zoppino, F.C.; Militello, R.D.; Slavin, I.; Alvarez, C.; Colombo, M.I., Autophagosome formation depends on the small GTPase Rab1 and functional ER exit sites. *Traffic* **2010**, *11*, 1246–1261.
2. Szatmari, Z.; Sass, M., The autophagic roles of Rab small GTPases and their upstream regulators: A review. *Autophagy* **2014**, *10*, 1154–1166.
3. Li, L.; Kim, E.; Yuan, H.; Inoki, K.; Goraksha-Hicks, P.; Schiesher, R.L.; Neufeld, T.P.; Guan, K.L., Regulation of mTORC1 by the Rab and Arf GTPases. *J. Biol. Chem.* **2010**, *285*, 19705–19709.
4. Talaber, G.; Miklossy, G.; Oaks, Z.; Liu, Y.; Tooze, S.A.; Chudakov, D.M.; Banki, K.; Perl, A., HRES-1/Rab4 promotes the formation of LC3(+) autophagosomes and the accumulation of mitochondria during autophagy. *PLoS ONE* **2014**, *9*, e84392.
5. Su, W.C.; Chao, T.C.; Huang, Y.L.; Weng, S.C.; Jeng, K.S.; Lai, M.M. Rab5 and class III phosphoinositide 3-kinase Vps34 are involved in hepatitis C virus NS4B-induced autophagy. *J. Virol* **2011**, *85*, 10561–10571, doi:10.1128/JVI.00173-11.
6. Krishnan, M.N.; Sukumaran, B.; Pal, U.; Agaisse, H.; Murray, J.L.; Hodge, T.W.; Fikrig, E. Rab 5 is required for the cellular entry of dengue and West Nile viruses. *J. Virol* **2007**, *81*, 4881–4885, doi:10.1128/JVI.02210-06.
7. Maday, S.; Wallace, K.E.; Holzbaur, E.L., Autophagosomes initiate distally and mature during transport toward the cell soma in primary neurons. *J. Cell Biol.* **2012**, *196*, 407–417.
8. Hyttinen, J.M.; Niittykoski, M.; Salminen, A.; Kaarniranta, K. Maturation of autophagosomes and endosomes: A key role for Rab7. *Biochim. Biophys. Acta* **2013**, *1833*, 503–510, doi:10.1016/j.bbamcr.2012.11.018.
9. Yu, L.; McPhee, C.K.; Zheng, L.; Mardones, G.A.; Rong, Y.; Peng, J.; Mi, N.; Zhao, Y.; Liu, Z.; Wan, F.; et al. Termination of autophagy and reformation of lysosomes regulated by mTOR. *Nature* **2010**, *465*, 942–946.
10. Lin, W.J.; Yang, C.Y.; Li, L.L.; Yi, Y.H.; Chen, K.W.; Lin, Y.C.; Liu, C.C.; Lin, C.H., Lysosomal targeting of phafin1 mediated by Rab7 induces autophagosome formation. *Biochem. Biophys Res. Commun.* 2012, 417, (1), 35- 42.
11. Flinn, R.J.; Yan, Y.; Goswami, S.; Parker, P.J.; Backer, J.M., The late endosome is essential for mTORC1 signaling. *Mol. Biol. Cell* 2010, 21, 833– 41.
12. Dupont, N.; Jiang, S.; Pilli, M.; Ornatowski, W.; Bhattacharya, D.; Deretic, V. Autophagy-based unconventional secretory pathway for extracellular delivery of IL-1beta. *EMBO J.* **2011**, *30*, 4701–4711, doi:10.1038/emboj.2011.398.
13. Pilli, M.; Arko-Mensah, J.; Ponpuak, M.; Roberts, E.; Master, S.; Mandell, M.A.; Dupont, N.; Ornatowski, W.; Jiang, S.; Bradfute, S.B.; et al. TBK-1 promotes autophagy-mediated antimicrobial defense by controlling autophagosome maturation. *Immunity* **2012**, *37*, 223–234.
14. Xu, X.F.; Chen, Z.T.; Zhang, J.L.; Chen, W.; Wang, J.L.; Tian, Y.P.; Gao, N.; An, J., Rab8, a vesicular traffic regulator, is involved in dengue virus infection in HepG2 cells. *Intervirology* **2008**, *51*, 182–188.
15. Nozawa, T.; Aikawa, C.; Goda, A.; Maruyama, F.; Hamada, S.; Nakagawa, I., The small GTPases Rab9A and Rab23 function at distinct steps in autophagy during Group A Streptococcus infection. *Cell Microbiol.* **2012**, *14*, 1149–1165.
16. Nishida, Y.; Arakawa, S.; Fujitani, K.; Yamaguchi, H.; Mizuta, T.; Kanaseki, T.; Komatsu, M.; Otsu, K.; Tsujimoto, Y.; Shimizu, S., Discovery of Atg5/Atg7-independent alternative macroautophagy. *Nature* **2009**, *461*, 654–658.
17. Puri, C.; Renna, M.; Bento, C.F.; Moreau, K.; Rubinsztein, D.C., Diverse autophagosome membrane sources coalesce in recycling endosomes. *Cell* **2013**, *154*, 1285–1299.
18. Szatmari, Z.; Kis, V.; Lippai, M.; Hegedus, K.; Farago, T.; Lorincz, P.; Tanaka, T.; Juhasz, G.; Sass, M., Rab11 facilitates cross-talk between autophagy and endosomal pathway through regulation of Hook localization. *Mol. Biol. Cell* **2014**, *25*, 522–531.
19. Tang, W.C.; Lin, R.J.; Liao, C.L.; Lin, Y.L. Rab18 facilitates dengue virus infection by targeting fatty acid synthase to sites of viral replication. *J. Virol* **2014**, *88*, 6793–6804, doi:10.1128/JVI.00045-14.
20. Munafo, D.B.; Colombo, M.I., Induction of autophagy causes dramatic changes in the subcellular distribution of GFP-Rab24. *Traffic* **2002**, *3*, 472–482.
21. Hirota, Y.; Tanaka, Y., A small GTPase, human Rab32, is required for the formation of autophagic vacuoles under basal conditions. *Cell Mol. Life Sci.* **2009**, *66*, 2913–2932.
22. Itoh, T.; Fujita, N.; Kanno, E.; Yamamoto, A.; Yoshimori, T.; Fukuda, M., Golgi-resident small GTPase Rab33B interacts with Atg16L and modulates autophagosome formation. *Mol. Biol. Cell* **2008**, *19*, 2916–2925.
